# A Safe Heuristic Path-Planning Method Based on a Search Strategy

**DOI:** 10.3390/s24010101

**Published:** 2023-12-24

**Authors:** Xiaozhen Yan, Xinyue Zhou, Qinghua Luo

**Affiliations:** 1 School of Information Science and Engineering, Harbin Institute of Technology at WeiHai, No. 2 Wenhua West Road, Weihai 264209, China; yanxiaozhen@hit.edu.cn (X.Y.); zxyuelu@163.com (X.Z.); 2 Shandong Institute of Shipbuilding Technology, Ltd., Weihai 264209, China

**Keywords:** mobile robot, path planning, collision-free safety, optimal boundary, heuristics

## Abstract

In industrial production, it is very difficult to make a robot plan a safe, collision-free, smooth path with few inflection points. Therefore, this paper presents a safe heuristic path-planning method based on a search strategy. This method first expands the scope of the search node, then calculates the node state based on the search strategy, including whether it is a normal or dangerous state, and calculates the danger coefficient of the corresponding point to select the path with a lower danger coefficient. At the same time, the optimal boundary is obtained by incorporating the environmental facilities, and the optimal path between the starting point, the optimal boundary point and the end point is obtained. Compared to the traditional A-star algorithm, this method achieved significant improvements in various aspects such as path length, execution time, and path smoothness. Specifically, it reduced path length by 2.89%, decreased execution time by 13.98%, and enhanced path smoothness by 93.17%. The resulting paths are more secure and reliable, enabling robots to complete their respective tasks with reduced power consumption, thereby mitigating the drain on robot batteries.

## 1. Introduction

As an intelligent system with advanced technology, an unmanned system can independently perform complex tasks without human intervention. In various unmanned systems, mobile robots, as an important part of life and work, have been widely used in industrial manufacturing, logistics, and medical fields [1]. Path planning, as a key technology for finding a safe and collision-free optimal path from the starting point to the target point in a given obstacle environment, has an important impact on the function and performance of unmanned systems. For example, it can help robots transport materials faster and more safely and complete the handling of goods and other operations [2]. Autonomous robots can reduce human work in the evacuation process and guide people to the best path to an exit [3]. Paddy field seeding robots improve the quality and efficiency of human work [4]. In these application scenarios, mobile robots often need to perform tasks in complex environments, such as narrow canals, factory workshops, or hospital corridors, so it is particularly important to ensure the safety of the robot.

As computer technology and artificial intelligence continue to advance, the field of path planning is evolving as well. From the earliest rule-based path-planning methods to research in path planning that incorporates graph theory [5], search algorithms [6], neural networks [7], and deep reinforcement learning [8], these technologies have driven continuous improvements in path planning. For example, in reference [9], the authors introduced a directional guidance strategy on top of traditional Rapidly exploring Random Trees (RRTs), simplifying invalid paths through linear processing and optimizing threshold parameters to balance the path length and planning time. Simulation experiments validated the algorithm’s superiority. Reference [10] combines the strengths of the A-star algorithm and interpolation algorithms, optimizing the A-star algorithm through geometric rules. It introduces the geometric A-star algorithm, which applies filtering functions to the paths generated by the A-star algorithm to address issues like excessive turning angles and irregular paths. Reference [11] introduces a mobile robot path-planning algorithm that combines the use of water flow potential fields and the beetle antennae search. This method divides the global path into multiple segments by using beetle genetic algorithms to set segmented waypoints. It employs a natural water flow approach for obstacle avoidance routing and optimizes the station coordinates using the beetle antennae search algorithm, thereby enhancing path quality. In reference [12], a hybrid solution method for mobile robot path planning is proposed, combining the ant colony optimization and artificial bee colony algorithms (IACO-IABC). This method reduces the number of turns in the planned path by incorporating three mechanisms within the IACO-IABC algorithm.

During task execution, the robot’s energy consumption significantly affects its operational time and performance. Therefore, energy conservation is crucial. To achieve energy efficiency and environmental friendliness during robot task execution, researchers have integrated data modeling, optimization algorithms, and neural networks into path-planning algorithms. For instance, reference [13] considers the discharging process of lithium batteries and converts the robot’s motion energy consumption into the occupancy time of a path grid. Reference [14] utilizes an energy consumption estimation model to estimate the energy consumption between neighboring positions and the destination using distance integration estimation models, combining both models to determine the path cost. Simulation experiments demonstrated that the proposed method generates optimal paths, saving a substantial amount of energy. Reference [15] takes into account different terrain conditions and turning angles as constraint factors. It adjusts the heuristic function weight of the A-star algorithm to reduce energy consumption in complex environments.

Furthermore, path smoothness significantly impacts robot motion. Path smoothing is vital for handling robots, as it not only reduces energy consumption and improves motion stability but also extends the equipment’s lifespan. For example, in reference [16], adaptive fractional velocity is introduced within a particle swarm algorithm to enhance its capability to escape local minima, while simultaneously employing continuous high-order Bézier curves to smooth the robot’s path. Improved particle swarm optimization is validated through simulation experiments. Reference [17] proposes a local block path-planning method, which utilizes intersection points between straight lines and obstacles as centers for finding intersections between lines and circles. These two intersection points serve as local starting and target points, followed by employing the A-star algorithm to find the optimal path. Finally, the straight lines and local paths are merged. Experiments show that this method solves the issue of excessive path reversal caused by the A-star algorithm. Reference [18] introduces a path-planning method based on cubic spline interpolation to maintain the smoothness of robot motion paths. The control points of the path, including the start point, control points, and destination point, are interpolated to form a complete path. Chaotic adaptive particle swarm optimization is used to optimize the control points, and the simulation results confirm the algorithm’s effectiveness. Reference [19] emphasizes the importance of smoothness in robot motion and combined genetic algorithms with B-spline curve generation techniques to create continuous obstacle avoidance curves.

The aforementioned research has introduced a variety of methods into the field of robot path planning, expanding the applicability of robots. However, as path planning constitutes a pivotal component of robot motion control, it necessitates a more comprehensive evaluation system to ensure the generation of higher-quality paths. It is worth noting that the aforementioned studies often focus on singular or limited factors, failing to fully consider the multidimensional aspects of path assessment. Particularly in complex environments, leveraging environmental features can aid in generating more rational path-planning solutions. Yet, the previous research has not delved deeply into this aspect, neglecting elements such as environmental infrastructure. This study adopts a holistic approach, taking into account several critical factors, including safety, path feasibility, and energy consumption. By introducing heuristic functions like the risk coefficient, it effectively enhances the accuracy and efficiency of path planning.

Our research primarily focuses on warehouse handling robots, with a specific emphasis on their application in warehousing environments. Within warehouses, numerous shelves pose as implicit obstacles for robots. Therefore, ensuring that robots can navigate accurately through these shelves and reach designated storage locations requires the design of an effective path. We particularly address the performance requirements of paths in the storage areas of warehouses, dedicating our research efforts to optimizing these performances. The goal is to obtain a more applicable path-planning solution for warehouse environments.

Within the warehouse milieu, the pivotal role of handling robots lies in their capacity to concurrently elevate logistical and handling efficiency while minimizing the burdens associated with manual operations. For the robot, multiple shelves within the warehouse serve as potential impediments. Consequently, ensuring that the robot reaches its designated shelf without encountering collisions with others becomes imperative, establishing a prerequisite for the secure storage of goods and the expeditious selection of items. Moreover, precise path planning contributes to the reduction in the robot’s movements within the warehouse, thereby augmenting the overall operational efficiency. Through the optimization of paths, the robot’s travel distance can be diminished, consequently lowering energy consumption and extending both the operational time and battery lifespan of the robot.

In response to these requirements, this paper introduces a safe heuristic path-planning method based on a search strategy. What sets this method apart is its ability to evaluate the danger coefficient of search nodes during the path-planning process, enabling the selection of safe, collision-free paths while reducing unnecessary turning points for smoother paths. Through this method, the energy consumption of handling robots is successfully reduced. Moreover, by considering the characteristics of the environmental facilities, the method achieves optimal boundary identification, resulting in the determination of the best path from the starting point to the optimal boundary point and then to the destination point.

The remainder of this article is organized as follows. Section 2 introduces the safe heuristic path-planning method. Section 3 presents the experimental simulation environment and performance evaluation. Section 4 gives the simulation results and a discussion of the system, and Section 5 summarizes this paper.

## 2. Methods

In this section, we first explain the framework of the method proposed in this paper. Then, the details of each submodule are presented, including the theoretical basis and implementation details.

### 2.1. Safe Heuristic Framework Based on a Search Strategy

**Figure 1:**
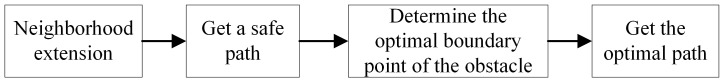
Framework of the safe heuristic path-planning method based on the search strategy.

The framework of the safe heuristic path-planning method based on a search strategy is shown in Figure 1. It mainly consists of three parts: neighborhood expansion, path selection using the hazard coefficient, and the determination of the optimal boundary points of obstacles. First, the A-star search algorithm is extended to obtain multi-neighborhood search nodes. Then, the hazard coefficient of each node is calculated to select a safe and effective path. Finally, according to the environmental information, the optimal boundary point is obtained, and the optimal path between the starting point, the optimal boundary point and the end point is obtained by using the safe heuristic method.

Neighborhood expansion: Due to the low search freedom of the A-star algorithm, the planned path has many turning points, so we expand the search neighborhood.

Path selection with the danger coefficient: According to the corresponding location information of the expanded node, the danger coefficient is calculated, and the node with the lower danger coefficient is selected as the specific path point.

Determination of the optimal boundary point: The starting point and the end point are connected to obtain a straight line segment. The straight line segment is intersected with the obstacle to obtain the intersection point, and the boundary point set of the intersecting obstacle is obtained. The distance between each boundary point and the intersection point is used as a criterion to find the optimal boundary point; the improved algorithm is used to find the local path between the starting point, the optimal boundary point and the end point. We combine the respective local paths as a global path.

### 2.2. Neighborhood Extension

The traditional A-star algorithm usually selects the point with the smallest evaluation function value as the next computation node [20] to optimize the search path. The evaluation function is expressed as:

(1) f(n)=g(n)+h(n)

In the formula, f(n) represents the total travel cost from the current node to the destination node; g(n) represents the actual travel cost from the current node to the starting node; and h(n) represents the estimated travel cost from the current node to the destination node. The Manhattan distance, Euclidean distance, and Chebyshev distance are commonly used to calculate h(n) [21]. The corresponding formulas are shown in Equations (2)–(4):

(2) $$h_{M}\left(n\right)=\left|x_{n}-x_{goal}\right|+\left|y_{n}-y_{goal}\right|$$

(3) $$h_{E}\left(n\right)=\sqrt{{\left(x_{n}-x_{goal}\right)}^{2}+{\left(y_{n}-y_{goal}\right)}^{2}}$$

(4) $$h_{C}\left(n\right)=\max\left\{\left.\left|x_{n}-x_{goal}\right|,\left|y_{n}-y_{goal}\right|\right\}\right.$$

where $h_{M}\left(n\right)$, $h_{E}\left(n\right)$, and $h_{C}\left(n\right)$ represent the heuristic functions obtained by using the Manhattan distance, Euclidean distance and Chebyshev distance, respectively; $\left(x_{n},y_{n}\right)$ are the coordinates of the current node; and $\left(x_{goal},y_{goal}\right)$ are the coordinates of the target node.

**Figure 2:**
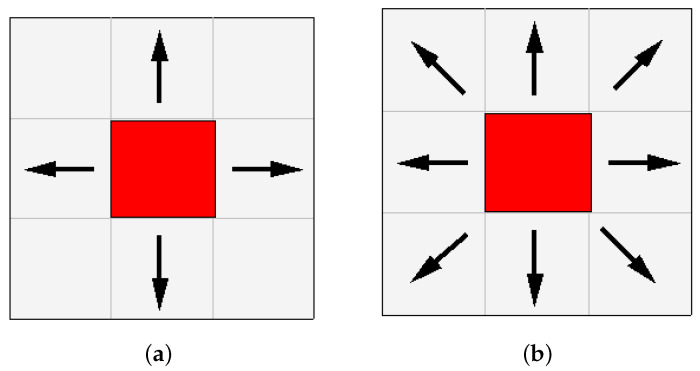
(**a**) Manhattan distance search directions, (**b**) Euclidean distance search directions.

When the A-star algorithm employs the Manhattan distance for path exploration, the degrees of freedom in search are limited to four directions—namely, upwards, downwards, leftwards, and rightwards—from the current node. Conversely, the A-star algorithm utilizing the Euclidean distance can discern one of eight adjacent nodes as the next search direction as illustrated in Figure 2. From Figure 2a, it is evident that the Manhattan distance search directions exhibit a 45° angle at each corner, thereby restricting exploration along the diagonal directions and introducing constraints on optimal paths involving certain diagonal movements. In Figure 2b, the Euclidean distance search directions feature a 22.5° angle at each corner, taking into account the diagonal directions and thereby fostering a greater array of possibilities in the search outcomes. The Chebyshev distance defines the distance between two points as the maximum absolute difference between their respective coordinate values. It calculates the number of moves required from the current node to the target node, commonly applied in highly specific scenarios such as warehouse and logistics environments. Consequently, the utilization of the Chebyshev distance as a general distance metric proves challenging.

**Figure 3:**
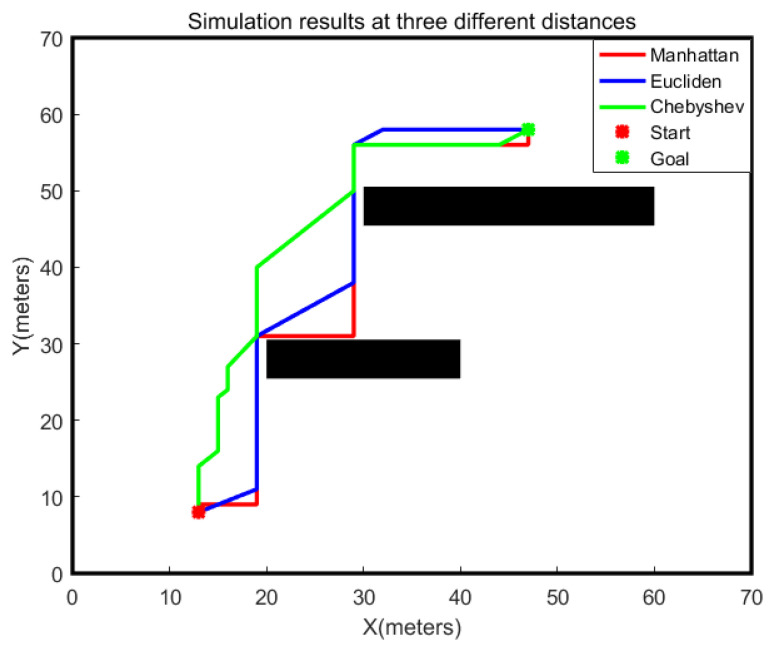
Comparison of the paths obtained by different distance measures.

The paths planned by the A-star algorithm using three different distances are shown in Figure 3. Given the same start and end points, the red path is planned by the A-star algorithm using the Manhattan distance, the blue path is planned using the Euclidean distance, and the green path is planned using the Chebyshev distance. The corresponding path lengths are shown in Table 1. According to the planned paths, it can be seen that the path obtained by the A-star algorithm using the Euclidean distance is shorter, and the turning point is smaller. Additionally, to be more consistent with the actual distance when the robot moves, this paper adopts the Euclidean distance.

**Figure 4:**
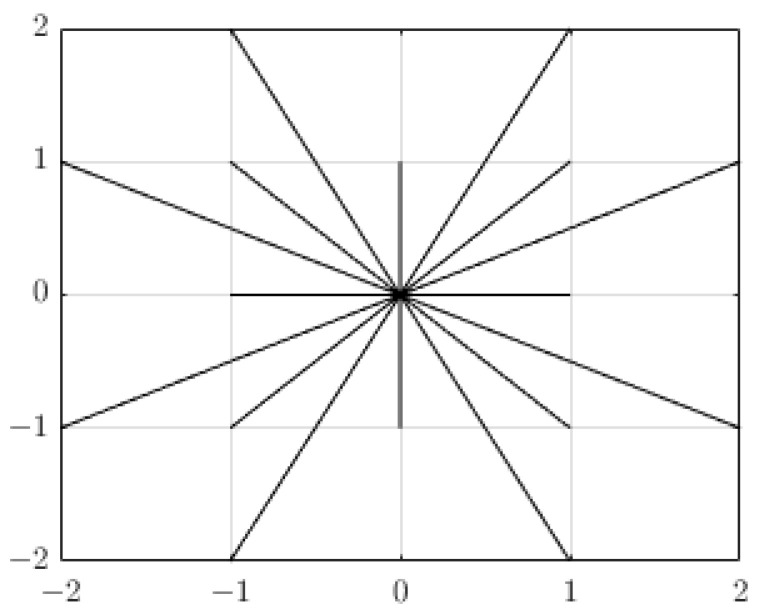
Extended 16-neighborhood search.

It can be seen from Figure 2b that the Euclidean distance uses an 8-neighborhood search; that is, there is an angle of 45° in each direction. When searching in this way, the distance between each search direction point and the current node is relatively singular. This single search method limits the planning of the path, and it is easy to obtain too many turning points in the final planned path as well as an insufficiently smooth trajectory. To address these problems, this paper presents an extended 16-neighborhood search method as shown in Figure 4. It can be seen from the figure that the angle of the extended search direction is 22.5°; the distance between the current node and the search node that is used in the traditional search method is too restricted. In the proposed method, the distance is diversified, and the degrees of freedom of the search increase, so the planned path is smoother and better, reducing unnecessary turning points and power consumption.

### 2.3. Selecting the Path by Using the Risk Coefficient

**Figure 5:**
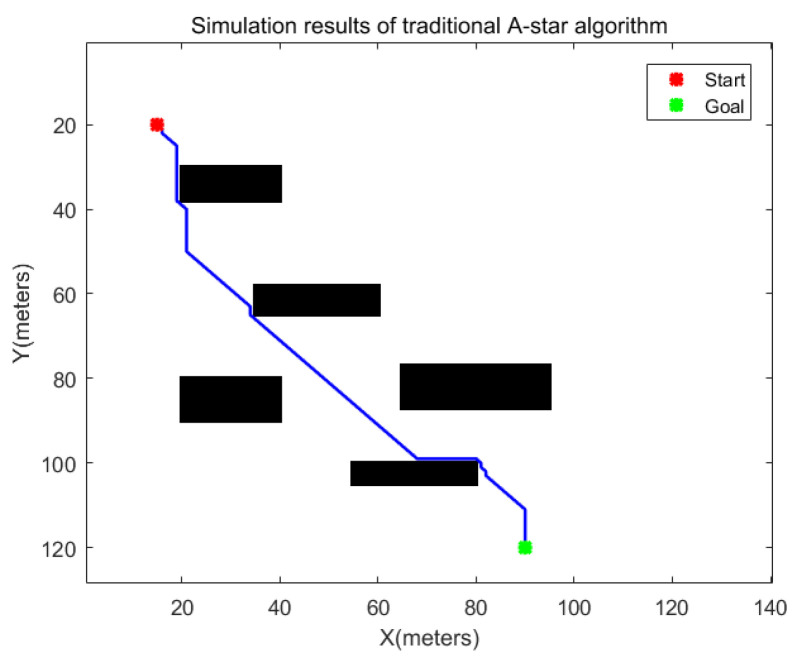
The path of the traditional A-star algorithm when passing obstacles.

In a real scene, the movement of the robot must not only consider the shortest path but also ensure that the path is safe and effective. As shown in Figure 5, when calculating the optimal path, the A-star search algorithm generates a path that is too close to the obstacle when passing the obstacle, which is not feasible in actual production and logistics. In addition, the robot itself has a certain shape and size, so it is necessary to consider the safety factors and dynamic constraints of the robot when planning the path.

To address these problems, this paper presents an improved scheme. Comprehensively considering various factors such as robot kinematics constraints, environmental obstacle information, and objective functions, it is assumed that the internal radius of the robot is $r_{robot}$, and *R* represents the distance between the robot and the obstacle. Considering the safety of the robot path, the minimum safe distance $R_{1}=1.5r_{robot}$ is set. If the distance between a node in the search environment and an obstacle is less than the minimum safe distance, we determine that the node is in a dangerous state and calculate the corresponding danger coefficient. The formula of the danger coefficient is as follows:

(5) $$\psi =\left\{\begin{array}{cc} 0 & R>R_{1}\\ \sum_{i=1}^{n}{\left(\frac{q}{\sqrt{{\left(x_{n}-x_{obsi}\right)}^{2}+{\left(y_{n}-y_{obsi}\right)}^{2}}}\right)}^{C} & R\leq R_{1} \end{array}\right.$$

where ψ sets the hazard coefficient between the robot and the obstacle, $\left(x_{obsi},y_{obsi}\right)$ is the coordinate of the obstacle whose distance from the robot is less than the minimum safe distance, *q* sets the affected area according to the physical size of the obstacle, and the effective range of the obstacle is determined by the positive integer *C*. The general parameters *q* and *C* are usually set to 2 and 1, respectively. The hazard coefficient is added to the heuristic function to generate a safer and more effective path-planning scheme. We change the heuristic function to:

(6) $$h\left(n\right)=h_{E}\left(n\right)+\psi$$

Algorithm 1 shows the pseudocode for the extended A-star algorithm, which takes the robot’s start point, target point, and map information as input and generates the optimized path best_path as output.
**Algorithm 1** Improved A-star algorithm  1:**function** A-STAR(start,goal,map)  2:   close←NULL  3:   open←start  4:   G[start]←g[start]=0,H[start]←h[start]  5:   F[start]←H[start]  6:   **while** open≠∅ **do**  7:      neighbor_Node← Select the node with the lowest F value in the open list  8:      open←open∖{current_Node}  9:      close←close∪{current_Node}10:      **if** current_Node=goal **then**11:         **return** best_path12:      **end if**13:      current_Node← Compute the set of vertices surrounding the current vertex14:      **for** N∈neighbor_Nodes **do**15:         **if** N∈close **then**16:            do nothing17:         **else if** N∈open **then**18:            G[N_new_calculated],H,F← Calculate the G, H, and F values of N19:            **if** G[N∈open]>G[N_new_calculated] **then**20:               Parent node of N ←current_Node21:            **end if**22:         **else**23:            Parent node of N ←current_Node24:         **end if**25:      **end for**26:   **end while**27:**end function**

### 2.4. Calculating the Optimal Boundary Point

In this paper, the idea of local environment block planning is used to analyze the environment information based on the principle of the shortest two-point line segments, and the boundary points of obstacles connecting the starting point and the end point are obtained. Local path planning is performed between the starting point, the boundary point and the end point, and then the local paths are merged. The principle of calculating the optimal boundary point is introduced below.

Suppose the start point in the environmental information is $A(x_{start},y_{start})$, the end point is $B(x_{goal},y_{goal})$, and the start and end points are connected. The line $\bar{AB}$ is obtained, and the equation of the analytic geometry line $\bar{AB}$ is:

(7) $$\left(y-y_{start}\right)\left(x_{goal}-x_{start}\right)=\left(x-x_{start}\right)\left(y_{goal}-y_{start}\right)$$

**Figure 6:**
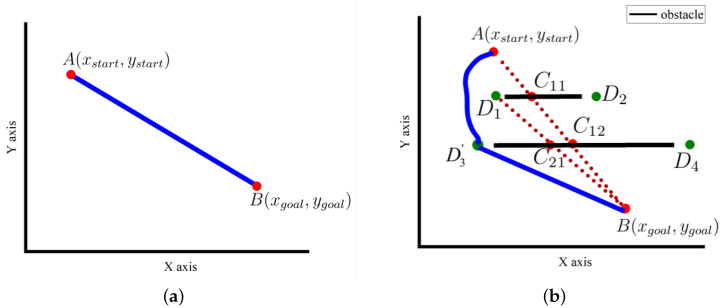
Algorithm planning path schematic diagrams. (**a**) Obstacle-free path, (**b**) path with obstacles.

Assuming that the line segment $\bar{AB}$ does not pass through an obstacle, the shortest path planned by the starting point and the destination point is the line segment $\bar{AB}$ shown in Figure 6a.

If the line segment $\bar{AB}$ passes through an obstacle, for the sake of simplicity, we assume that the number of obstacles passed through is 2 as shown in Figure 6b. First, the intersection points $C_{1}$ and $C_{2}$ of the obstacle and the line $\bar{AB}$ are obtained. Using the first intersection $C_{1}\left(x_{C_{1}},y_{C_{1}}\right)$, the boundary point $D_{i}\left(x_{D_{i}},y_{D_{i}}\right)\left(i=1,\;2,\;3,\cdots \right)$ of the first obstacle is obtained. We calculate the distance between the boundary point and the intersection $C_{1}\left(x_{C_{1}},y_{C_{1}}\right)$:

(8) $$d_{i}=\sqrt{{\left(x_{C_{1}}-x_{D_{i}}\right)}^{2}+{\left(y_{C_{1}}-y_{D_{i}}\right)}^{2}}$$

**Figure 7:**
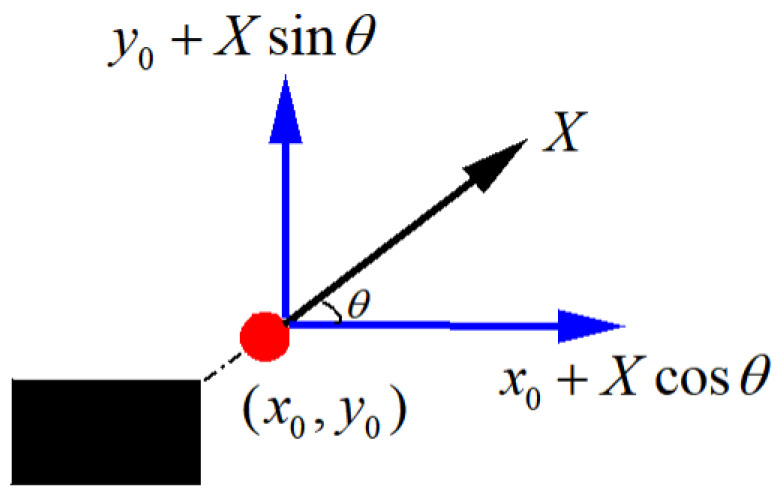
Obtaining a new boundary point.

We find the boundary point corresponding to the minimum distance. In Figure 6b, this point is $D_{1}\left(x_{D_{1}},y_{D_{1}}\right)$. We abandon $D_{2}\left(x_{D_{2}},y_{D_{2}}\right)$, take $D_{1}\left(x_{D_{1}},y_{D_{1}}\right)$ as a new starting point, connect the starting point and the end point, obtain the line $\bar{D_{1}B}$, find the intersection coordinate $C_{3}\left(x_{C_{3}},y_{C_{3}}\right)$ of the obstacle passing through the line segment $\bar{D_{1}B}$, obtain the boundary point of the obstacle corresponding to the first intersection, calculate the distance from the intersection $C_{3}\left(x_{C_{3}},y_{C_{3}}\right)$, and find the optimal boundary point corresponding to the minimum distance $D_{3}\left(x_{D_{3}},y_{D_{3}}\right)$ in the figure. Using this point as a new starting point, we continue to connect the starting point to the target point, obtain the line segment $\bar{D_{3}B}$, and determine whether the line passes through the obstacle. Considering that the robot itself has a certain shape and size, the distance between the border point and the obstacle cannot be too small. Assuming that the border point is $E(x_{0},y_{0})$, the safe distance is also set to $R_{1}$, and an obstacle whose distance from $E(x_{0},y_{0})$ is less than the safe distance is set as a threatening obstacle. The formula of the corresponding obstacle coefficient *X* is shown in Equation (9):

(9) $$X=\sum_{i=0}^{n}\left(\frac{1}{\left|E-E_{obsi}\right|}-\frac{1}{R_{1}}\right)$$

where $E_{obsi}\;\left(i=0,\;1,\;\cdots ,\;n\right)$ represents the threatening obstacle encountered by the robot point $E(x_{0},y_{0})$, and $\left|E-E_{obsi}\right|$ represents the distance between the limit point and the obstacle; according to the obstacle coefficient, a new limit point $E^{{}^{\prime }}\left(x_{1},y_{1}\right)$ is obtained. The relationship between $E^{{}^{\prime }}\left(x_{1},y_{1}\right)$ and $E(x_{0},y_{0})$ is shown in Equation (10), and represents the angle between $E(x_{0},y_{0})$ and $E_{obs}$. The correspondence analysis is shown in Figure 7:

(10) $$\begin{array}{c} x_{1}=x_{0}+X\cos\theta \\ y_{1}=y_{0}+X\sin\theta \end{array}$$

In Figure 6b, it can be seen that straight line $\bar{D_{3}B}$ does not pass through the obstacle, and the last boundary point $D_{3}\left(x_{D_{3}},y_{D_{3}}\right)$ is maintained. The obstacle coefficient between $D_{3}\left(x_{D_{3}},y_{D_{3}}\right)$ and the obstacle is calculated, and the optimal boundary point $D_{3}^{{}^{\prime }}\left(x_{D_{3}},y_{D_{3}}\right)$ is obtained.

Taking the point $A(x_{start},y_{start})$ as the starting point and the optimal boundary point $D_{3}^{{}^{\prime }}\left(x_{D_{3}},y_{D_{3}}\right)$ as the destination point, the improved A-star algorithm is used for path planning to obtain local path 1. Then, taking the boundary point $D_{3}^{{}^{\prime }}\left(x_{D_{3}},y_{D_{3}}\right)$ as the starting point and the end point $B(x_{goal},y_{goal})$ as the destination point, according to the principle of the shortest line segment between the two points, path 2 is obtained, that is, the line segment $\bar{D_{3}^{{}^{\prime }}B}$. Combining the local paths, the optimal path from the starting point $A(x_{start},y_{start})$ to the destination point $B(x_{goal},y_{goal})$ is obtained, shown as the blue line in Figure 6b.

Algorithm 2 shows the pseudocode for obtaining the optimal border point. It takes as input the start point, target point, map and obstacle information of the robot and outputs the optimal border point as local_start.
**Algorithm 2** Obtain the best boundary point  1:**function** OBP(start,goal,map,obstacle)  2:   Line← Calculate the straight line from the start point to the end point  3:   cross_p,boundary_p,dis,local_stat←∅  4:   i←0  5:   **for** each obs∈obstacle **do**  6:      cross_p←ispass_line[obs,Line]  7:   **end for**  8:   **if** cross_p=∅ **then**  9:      local_start←start10:   **else**11:      boundary_p←boundary(cross_p(1))12:      **for** each boundary_p **do**13:         $dis\leftarrow {\left(boundary\_p-cross\_p\left(1\right)\right)}^{2}$14:      **end for**15:      **for** each dis **do**16:         i←find(dis=min(dis))17:      **end for**18:      local_start←boundary_p(i)19:   **end if**20:   **return** local_start21:**end function**

Algorithm 3 is the pseudocode for local path fusion. It takes as input the optimal border point, the target point, and the local path information of the robot, and outputs the optimal path as path.
**Algorithm 3** Merge local paths1:**function** Merge(goal_start,goal,best_path)2:   path1← Computes the line between the local_start and end points3:   path←[best_path;path1]4:   **return** path5:**end function**

### 2.5. Regularized Obstacles

In the case of irregular obstacles, due to their intricate shapes, mathematical modeling becomes exceedingly complex, particularly when confronted with the formidable challenge of identifying the model’s boundary points. To effectively address this issue, a series of strategies are employed in this study to streamline the process of handling irregular obstacles. The following provides a detailed description of these strategies.

**Figure 8:**
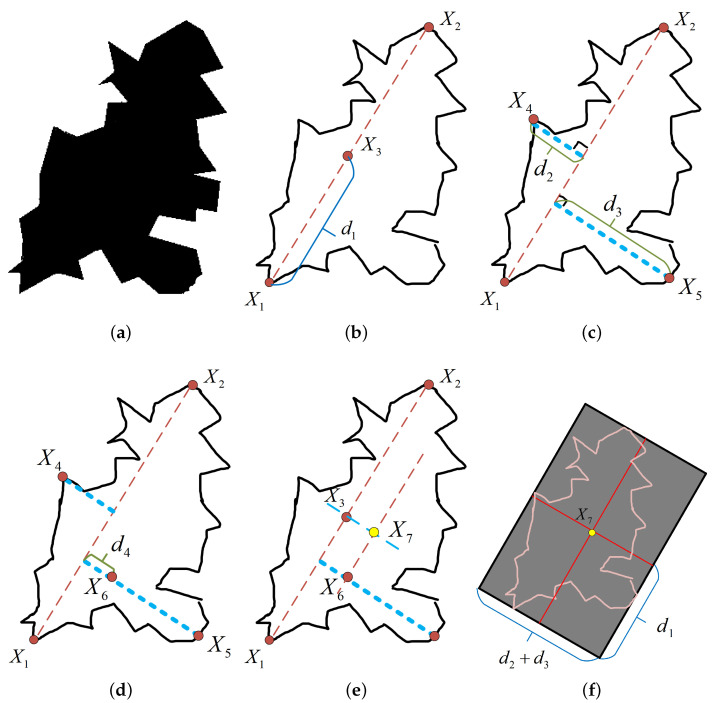
Steps for handling irregular obstacles. (**a**) Irregular obstacles, (**b**) find the midpoint $X_{3}$, (**c**) draw a perpendicular, (**d**) find the midpoint $X_{6}$ of the perpendicular, (**e**) find the center point $X_{7}$ of the obstacle, and (**f**) find the regularized obstacle.

As depicted in Figure 8a, we illustrate a typical irregular obstacle. Initially, we conduct a comprehensive analysis of the obstacle to extract its outer contour as demonstrated by the black line in Figure 8b. Subsequently, the search is initiated for the two farthest points on the outer contour, denoted as $X_{1}\left(x_{1},y_{1}\right)$ and $X_{2}\left(x_{2},y_{2}\right)$, with their connecting line referred to as $X_{1}$-$X_{2}$. This line’s equation is represented as per Equation (11), where *K* signifies the slope as specified in Equation (12). The midpoint $X_{3}\left(\frac{x_{2}+x_{1}}{2},\frac{y_{2}+y_{1}}{2}\right)$ between these two points is computed, and the distance from point $X_{3}$ to both points is defined as $d_{1}$ as portrayed in Figure 8b:

(11) $$\begin{array}{c} y=K\left(x-x_{1}\right)+y_{1} \end{array}$$

(12) $$\begin{array}{c} K=\frac{y_{2}-y_{1}}{x_{2}-x_{1}} \end{array}$$

The line $X_{1}$-$X_{2}$ effectively bifurcates the obstacle into left and right segments as delineated in Figure 8b on the left and right sides, respectively. Subsequently, we individually determine the maximum distances from each point on the outer contour to the line $X_{1}$-$X_{2}$ for both the left and right segments, as indicated by the blue lines in Figure 8c. These utmost distances are designated as $d_{2}$ and $d_{3}$, with corresponding points on the outer contour identified as $X_{4}\left(x_{4},y_{4}\right)$ and $X_{5}\left(x_{5},y_{5}\right)$. Assuming that $d_{3}$ surpasses $d_{2}$, a line segment designated as l1 is created, traversing through point $X_{5}\left(x_{5},y_{5}\right)$ and perpendicular to the line $X_{1}$-$X_{2}$, with its linear equation articulated in Equation (13), where the slope is $-\frac{1}{K}$:

(13) $$\begin{array}{c} y=-\frac{1}{K}\left(x-x_{5}\right)+y_{5} \end{array}$$

Moving forward, the quest to ascertain point $X_{6}\left(x_{6},y_{6}\right)$ ensues, situated at a distance of $d_{2}$ from $X_{5}\left(x_{5},y_{5}\right)$ and positioned on the line $l_{1}$ as elucidated in Figure 8d. At this juncture, the distance from $X_{6}\left(x_{6},y_{6}\right)$ to the line $X_{1}$-$X_{2}$ is determined as $d_{4}$, where $d_{4}=d_{3}-d_{2}$ as portrayed in Figure 8d. Subsequently, lines denoted as $l_{2}$ and $l_{3}$ are established, with $l_{2}$ having $X_{6}\left(x_{6},y_{6}\right)$ as the central point and a slope of *K*, while $l_{3}$ emanates from $X_{3}$ as the central point with a slope of $-\frac{1}{K}$. The intersection point of these two lines is identified as $X_{7}$ as illustrated in Figure 8e. Finally, with $X_{7}$ as the center point, we construct a rectangle with a length of $2d_{1}$ and a width of $d_{2}+d_{3}$, where the tilt angles of the two sides are arctan(K) and $\arctan(-\frac{1}{K})$, respectively, as shown in Figure 8f. This rectangular area represents a processed obstacle with a regular shape.

Through the employment of this strategic approach, we adeptly transform irregular obstacles into regular shapes, all the while ensuring that these regular-shaped obstacles wholly encapsulate the original irregular obstacles. This, in turn, serves to reduce the complexity associated with determining the boundary points of obstacles and effectively mitigates the risk of collisions with said obstacles.

## 3. Simulation Environment and Parameters

### 3.1. Evaluation Platform Configuration

This section introduces the map environment of robot path planning and its related settings, the robot parameters, the evaluation indicators and the simulation environment.

In this section, the mobile robot map environment is set up as a grid map with a length and width of 200 m. If the index value corresponding to a position in the grid map is 1, it indicates that there is an obstacle, and if the index value corresponding to a position in the grid map is 0, it indicates that there is no obstacle. The map layout is designed in two test scenarios. The computer configuration used in the simulation test experiment is the Windows 10 operating system, i5-7200U processor and 8 GB RAM, Dell Inc., Kunshan, China.

### 3.2. Scene Settings

In this paper, two different map layouts are created based on a flat ground design.

**Figure 9:**
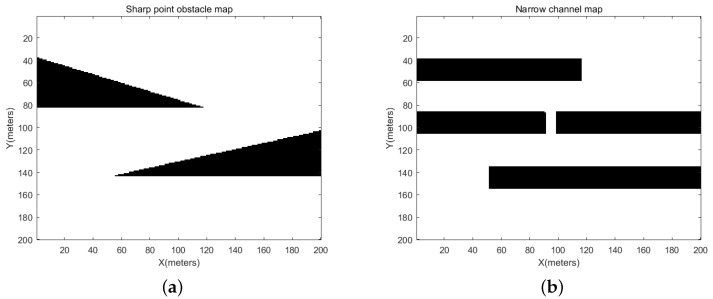
Maps for test scenario 1. (**a**) The environment of sharp obstacles, (**b**) the environment of narrow passages.

For the first type of environmental information, to better evaluate the performance of the proposed algorithm in terms of security and effectiveness, two different map environments are set up in this section. The map environment is shown in Figure 9. One map contains convex-tip obstacles, while the other map contains narrow channels. These different map environments are designed to examine whether the handling robot can safely navigate through these challenges to fully evaluate the algorithm. The robot’s starting position coordinates are set to (10,10), and the goods must be transported to the target position with coordinates (190,190).

For the second type of environmental information, to test the robustness of the algorithm in the case of random changes in the environmental information, this section presents an experiment that sets random changes in the obstacle environment. Specifically, three different maps are randomly generated, with obstacles accounting for 20% of each map. These randomly generated maps contain a combination of multiple obstacle locations to verify whether the algorithm can adapt to random changes in different environmental information. Through this experiment, the ability of the algorithm to detect and avoid randomly changing obstacle information can be evaluated, and the reliability and practicality of the algorithm can be further improved. The coordinates of the robot’s start position are set to (25,35), and the goods must be transported to the target position with coordinates (140,190).

### 3.3. Performance Indicators

**Figure 10:**
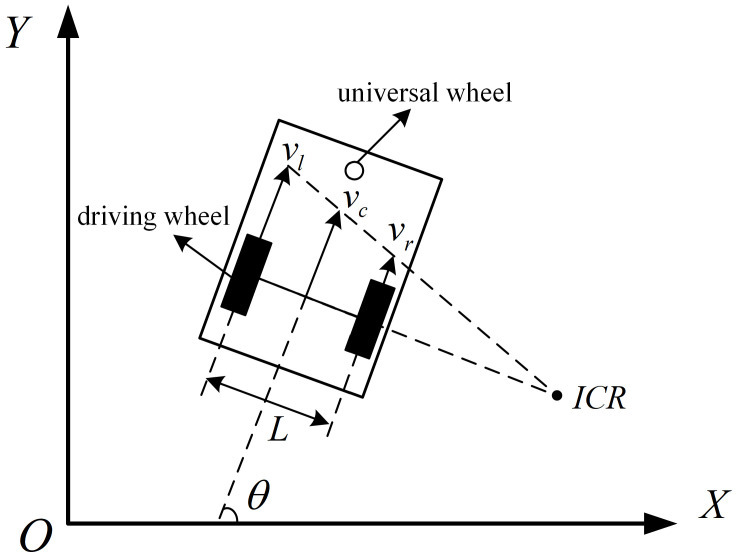
Schematic diagram of differential three-wheeled robot model.

In order to authentically assess the performance of the algorithm presented in this study, we adopt the differentially driven motion modelproposed in the referenced literature [22]. As illustrated in Figure 10, the robotic platform is equipped with an omnidirectional wheel and two drive wheels. In the model, $v_{l}$, $v_{r}$, and $v_{c}$ denote the linear velocities of the left drive wheel, right drive wheel, and overall robot, respectively. Symbol *L* represents the wheelbase between the left and right drive wheels. The robot’s angular velocity is denoted by $w_{c}$. Consequently, the kinematic equations governing the robot’s motion are expressed as follows:

(14) $$\begin{array}{c} \left(\begin{array}{c} v_{c}\\ \omega_{c} \end{array}\right)=\left(\begin{array}{cc} \frac{1}{2} & \frac{1}{2}\\ \frac{1}{L} & -\frac{1}{L} \end{array}\right)\left(\begin{array}{c} v_{r}\\ v_{l} \end{array}\right) \end{array}$$

(15) $$\begin{array}{c} \left(\begin{array}{c} {\dot{x}}_{c}\\ {\dot{y}}_{c}\\ \dot{\theta } \end{array}\right)=\left(\begin{array}{cc} \cos\theta & 0\\ \sin\theta & 0\\ 0 & 1 \end{array}\right)\left(\begin{array}{c} v_{c}\\ \omega_{c} \end{array}\right) \end{array}$$

Here, $x_{c}$, $y_{c}$, and θ respectively signify the velocities of the robot in the x-axis, y-axis, and angular directions.

**Figure 11:**
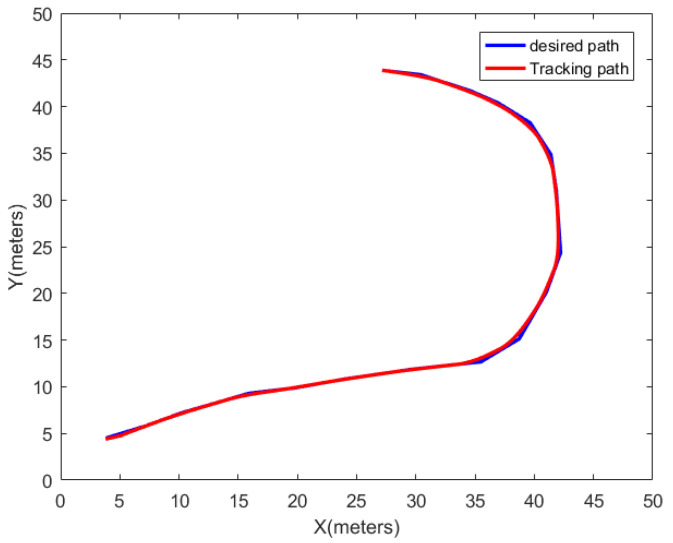
PID control differential two-wheeled robot-tracking path.

**Figure 12:**
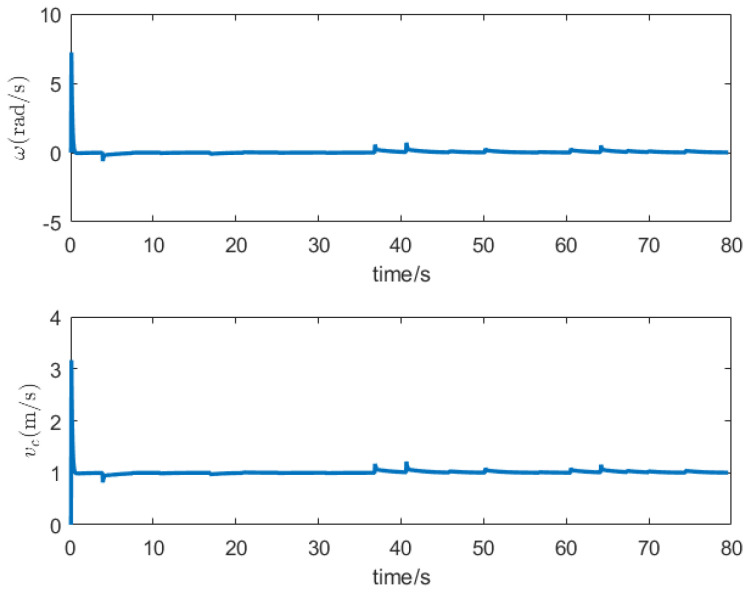
The angular velocity *w* and center velocity $v_{c}$ of the small car.

The PID controller stands as a classical feedback control methodology, continually adjusting the output to approximate the system’s actual output to the desired output [23]. Consequently, this manuscript employs the PID controller to govern the motion of a differentially driven two-wheeled vehicle, ensuring that the vehicle adheres to the planned trajectory. Under the assumption of a robot with a wheelbase of 60 cm and a travel speed of 1 m/s, the trajectory tracked by the differentially driven two-wheeled robot under PID control is illustrated in Figure 11. Figure 12 portrays the magnitudes of the robot’s angular velocity and center velocity on this trajectory.

The original path length is 73.73 m, and under PID control, the robot traverses this path in 79.4 s. Subsequently, this model is applied to path planning, and the simulated path is evaluated in the latter part of this paper.

The primary objective of the path planning endeavors to formulate an optimal collision-free trajectory. The consumption of electrical energy by the mobile robot serves as a pivotal determinant for the successful execution of its tasks. A superior trajectory holds the potential to abbreviate the robot’s traversal time, consequently mitigating power consumption. This, in turn, guarantees that the robot maintains an ample reserve of electrical energy to effectively accomplish its designated tasks, thereby augmenting the overall operational efficiency of the robot. Hence, it becomes imperative to mandate that the paths devised prioritize safety and smoothness while concurrently minimizing their length. This paper conducts a comprehensive assessment and discourse of the planned trajectories, evaluating and discussing them across six fundamental facets: path length, collision risk, path search duration, traversal duration, trajectory smoothness, and energy consumption.

(1) Path length

The goal is to achieve the shortest path possible. Assuming that the start position and target position are $S_{0}$ and $S_{1}$, respectively, the path length calculation formula is:

(16) $$\begin{array}{c} L\left(S\right)=\sum_{i=0}^{N}\left(S_{i+1}-S_{i}\right) \end{array}$$

where L(S) represents the path length and the unit is m. $S_{i+1}-S_{i}$ denotes the Euclidean distance between $S_{i+1}$ and $S_{i}$.

(2) Collision risk value

The collision risk value refers to the possibility of collision with the robot at a given point on the robot’s trajectory. If the robot collides while moving, it may cause equipment damage, mission failure, robot damage, or environmental pollution and may even cause injury to people or endanger human safety. Therefore, it is very important to evaluate and control the collision risk in robot motion control. In this paper, a two-dimensional Gaussian model is used to establish a collision safety function to estimate the collision safety value. The specific formula is as follows:

(17) $$\begin{array}{c} R\left(S_{robot},S_{obs}\right)=\sum_{i=0}^{n}e^{-0.5{\left(\frac{S_{robot}-S_{obsi}}{q}\right)}^{C}} \end{array}$$

where $S_{obs}$ represents an obstacle whose distance from the robot is less than the safe distance, and the general parameters *q* and *C* are usually set to 2 and 1, respectively.

(3) Exercise time

In practical tasks, the robot must complete the task on time, and when moving in a dangerous environment, shortening the time can reduce the risk of damage or danger to the robot. The time calculation formula is as follows:

(18) $$\begin{array}{c} T=\sum_{i=1}^{N-1}t_{i}=\sum_{i=1}^{N-1}\frac{V_{roboti}}{S_{i+1}-S_{i}} \end{array}$$

where $t_{i}$ is the movement time at the corresponding position $S_{i}$, $Y_{obs}$ represents the movement speed of the robot at position $S_{i}$, and the robot speed is as given above.

(4) Path smoothness

The smoother the planned path is, the fewer the turning points of the path, and the less energy and time consumed by the mobile robot. The smoothness of the path is mainly calculated by the turning angle of the mobile robot along the target path. The specific formula is

(19) $$\begin{array}{c} A\left(S\right)=\sum_{i=1}^{N}\gamma_{i}=\sum_{i=1}^{N}\arccos(\frac{\left(S_{i}-S_{i-1}\right)\cdot \left(S_{i+1}-S_{i}\right)}{\left|S_{i}-S_{i-1}\right|\times \left|S_{i+1}-S_{i}\right|}) \end{array}$$

where $\gamma_{i}$ is the value of the i-th corner of the obtained path (calculated in radians, varies from 0 to π), *N* is the number of points of the path motion of the mobile robot, and $\left(S_{i}-S_{i-1}\right)\cdot \left(S_{i+1}-S_{i}\right)$ is the inner product of two vectors.

(5) Power consumption

Robots with low power consumption can work more efficiently, reduce charging time and improve working efficiency. In this paper, it is assumed that the initial power of the robot is 100% in each test experiment, and when the 50 kg weight of the goods is carried, the robot will exhaust its power by moving 1000 m on a straight path. In the linear motion of the mobile robot, constant acceleration is used to control the speed to reduce the number of speed changes, which can effectively reduce the energy consumption. According to [24], the set speed changes every five time periods to consume 1% of the power. In addition, every time the rotation angle of the robot reaches 360°, it will consume 1% of the power. The formula for the quantity of power *E* remaining after the robot has completed the handling task is shown in Equation (20), where $N_{v}$ is the number of speed changes:

(20) $$\begin{array}{c} E=100\%-\left(\frac{L(s)}{1000}+\frac{N_{v}}{500}+\frac{A(s)}{200\pi }\right)\times 100\% \end{array}$$

In order to conduct a comprehensive evaluation of the algorithm, this study compares these metrics using percentages to better evaluate the performance of the algorithm. The calculation method is shown in Equations (21) to (26):

(21) $$P_{length}=\frac{length_{ref}-length_{pro}}{length_{ref}}\times 100\%$$

(22) $$P_{collision}=\frac{Collision_{ref}-Collision_{pro}}{Collision_{ref}}\times 100\%$$

(23) $$P_{S\_time}=\frac{S\_time_{ref}-S\_time_{pro}}{S\_time_{ref}}\times 100\%$$

(24) $$P_{R\_time}=\frac{R\_time_{ref}-R\_time_{pro}}{R\_time_{ref}}\times 100\%$$

(25) $$P_{smoothness}=\frac{Smoothness_{ref}-Smoothness_{pro}}{Smoothness_{ref}}\times 100\%$$

(26) $$P_{power}=\frac{\left(1-Power_{ref}\right)-\left(1-Power_{pro}\right)}{1-Power_{ref}}\times 100\%$$

In the equations, $length_{ref}$, $Collision_{ref}$, $S\_time_{ref}$, $R\_time_{ref}$, $Smoothness_{ref}$, and $Power_{ref}$ represent the path length, collision risk value, searching time, run time, smoothness, and residual power obtained by the reference methods, respectively. $length_{ref}$, $Collision_{ref}$, $S\_time_{ref}$, $R\_time_{ref}$, $Smoothness_{ref}$, and $Power_{ref}$ represent the corresponding metrics for the path planned by the algorithm proposed in this study. $P_{length}$, $P_{collision}$, $P_{S\_time}$, $P_{R\_time}$, $P_{smoothness}$, and $P_{power}$ represent the percentage reductions in path length, collision risk value, searching time, run time, smoothness, and residual power achieved by the algorithm proposed in this study compared to the reference methods.

## 4. Simulation Results and Discussion

This section discusses the feasibility and effectiveness of the safe heuristic algorithm based on the search strategy to design the map layout under different conditions. The proposed safe heuristic algorithm based on a search strategy (SHA-star), the traditional A-star algorithm [25] (T_A-star), the A-star algorithm after adding the danger coefficient in this paper [S_A-star], and the method of [17] (R_[17]) are taken as reference methods to evaluate the performance of the proposed algorithm. It is worth noting that the S_A-star algorithm does not originate from previous references but represents an enhancement built upon the foundation of the conventional A-star algorithm. This enhancement process involves the integration of the hazard coefficients introduced in this paper into the traditional A-star algorithm, aiming to seek optimal path planning while taking into full consideration the hazardous factors. The key objective of this approach is to verify whether the introduction of hazard coefficients, in comparison to the conventional A-star algorithm, effectively enhances the safety of robot path planning. To ensure the accuracy of the results, we repeated the same experiment 100 times and calculated the average of these repeated experiments.

### 4.1. Simulation Results and Discussion for the First Map Environment Category

**Figure 13:**
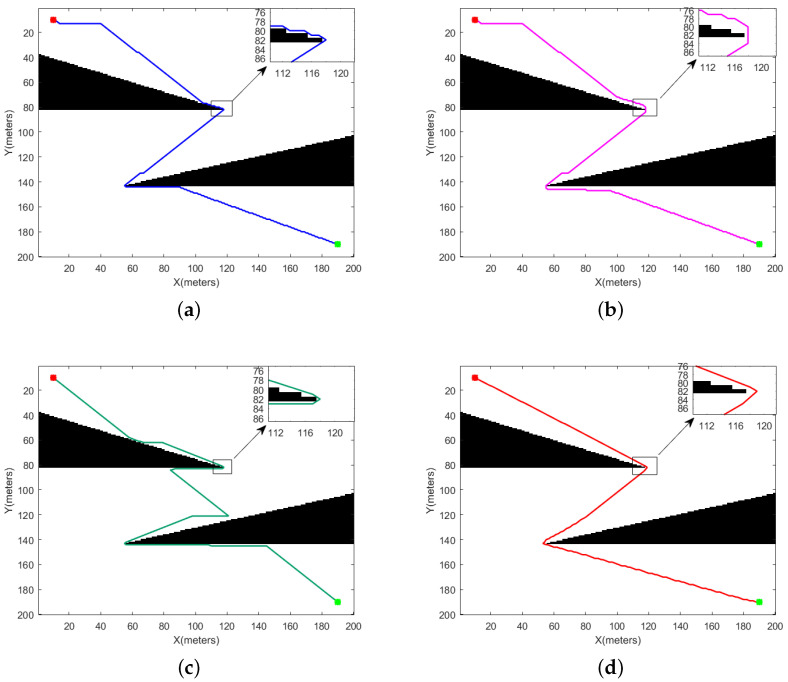
Path planning using different algorithms in sharp obstacle environments. (**a**) T_A-star, (**b**) S_A-star, (**c**) R_[17], (**d**) SHA-star.

**Figure 14:**
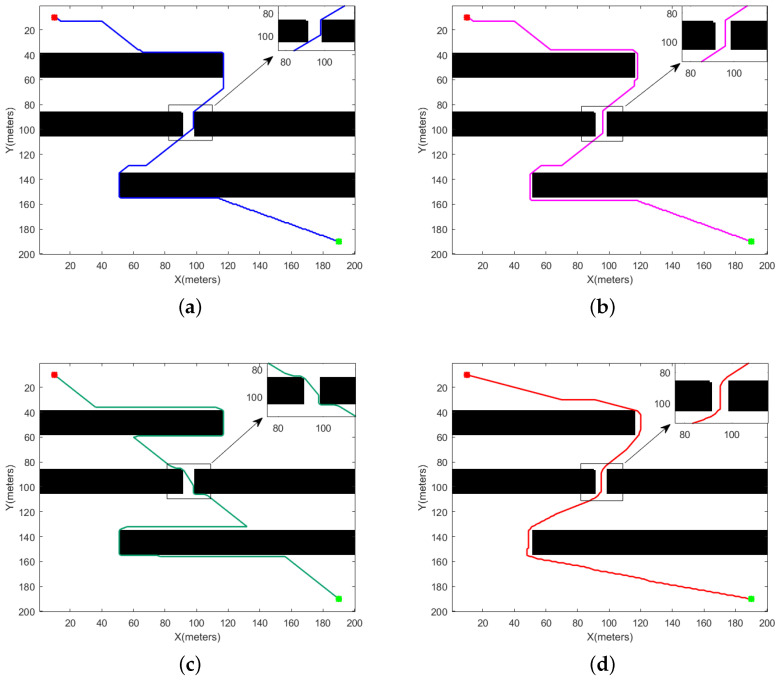
Planning results of different methods for a narrow channel. (**a**) T_A-star, (**b**) S_A-star, (**c**) R_[17], (**d**) SHA-star.

Figure 13 and Figure 14 delineate the simulation outcomes of the four algorithms within the first environmental category. The map denotes the starting point with red markers and the destination with green markers. Figure 13a–c and Figure 14a–c represent the results of the T_A-star, S_A-star, and R_[17] algorithms in the first environment, while Figure 13d and Figure 14d depict the paths derived by the SHA-star algorithm. It is conspicuous from the figures that all four algorithms successfully navigate without colliding with obstacles. The paths generated by SHA-star exhibit a notable safety margin from obstacles and remarkable smoothness. Table 2 encompasses a comprehensive analysis, comparing results in terms of path length, collision risk, search time, motion duration, path smoothness, and remaining power.

#### 4.1.1. Discussion on Convex Obstacles

The SHA-star algorithm amalgamates the local and linear paths, effectively reducing the path inflection points by increasing the search freedom. This dual effect results in shorter path lengths and enhanced path smoothness. In the context of convex obstacles, SHA-star excels with the optimal path length of 369.5521 and path smoothness of 6.1191. T_A-star and S_A-star adhere to the conventional 8-neighborhood method for pathfinding, which often leads to paths with numerous inflection points and suboptimal smoothness. Additionally, due to S_A-star’s need to select paths with lower danger coefficients, its optimal path length is slightly longer than that of T_A-star. Therefore, T_A-star’s optimal path length and path smoothness are 380.5584 and 89.5353, respectively, while S_A-star’s optimal path length and path smoothness are 384.0731 and 89.5353. R_[17], which leverages the A-star algorithm to find the shortest path between intersections of circles and lines, offering a hybrid approach that can reduce the path length in simpler environments. However, in complex environments, the path length significantly increases due to the time required to calculate these intersections. While R_[17] improves path smoothness by including straight-line segments, it does not consider collision risks. Consequently, R_[17] achieves an optimal path length of 446.3637 and a path smoothness of 15.3482 within convex obstacles.

Both SHA-star and S_A-star rely on danger coefficients to select safe paths, resulting in low collision risks. Specifically, in the context of convex obstacles, SHA-star and S_A-star exhibit collision risks of 10.6796 and 18.1278, respectively. T_A-star and R_[17], on the other hand, fail to account for path safety, leading to paths in close proximity to obstacles and high collision risks. Their collision risks within convex obstacles are 309.0022 and 674.88, respectively.

While both SHA-star and S_A-star continuously evaluate node danger coefficients, the former’s search time is increased, compared to T_A-star. However, SHA-star significantly reduces the search time for straight-line segments, resulting in shorter overall search times compared to S_A-star. R_[17], on the other hand, expends significant time in complex environments to find intersections between circles and lines, wasting search time. Nevertheless, the total runtime (total time = search time + motion duration) for SHA-star within convex obstacles is notably shorter than the other three algorithms. Specifically, SHA-star, T_A-star, S_A-star, and R_[17] exhibit total runtimes of 409.811 s, 414.515 s, 481.108 s, and 398.264 s, respectively.

Given that SHA-star’s path exhibits minimal length, enhanced smoothness, and low collision risks, it conserves energy within convex obstacles. Thus, in such scenarios, SHA-star retains a substantial power reserve of 62.07%, while T_A-star, S_A-star, and R_[17] have power reserves of 47.69%, 47.34%, and 52.92%, respectively. In conclusion, SHA-star excels in environments with convex obstacles.

#### 4.1.2. Discussion on Narrow Passages

The data in Table 2 reveal that in environments characterized by narrow passages, SHA-star outperforms T_A-star, S_A-star, and R_[17] in various performance aspects. Specifically, SHA-star reduces the path length by 1.74%, 2.93%, and 25.63% compared to T_A-star, S_A-star, and R_[17], respectively. It significantly mitigates collision risks by 98.79%, 89.7%, and 98.81%. Furthermore, it enhances path smoothness by 87.72%, 87.12%, and 56.65%. Energy consumption is reduced by 19.21%, 19.23%, and 27.24% when compared to T_A-star, S_A-star, and R_[17], respectively. Although SHA-star’s evaluation of node danger coefficients increases the search time compared to T_A-star, the total runtime (total time = search time + motion duration) for SHA-star is shorter. When considering the search time and runtime together, SHA-star outperforms S_A-star and R_[17] within the context of narrow passages.

In summary, SHA-star exhibits distinct superiority in environments characterized by narrow passages.

### 4.2. Simulation Results and Discussion for the Second Map Environment Category

**Figure 15:**
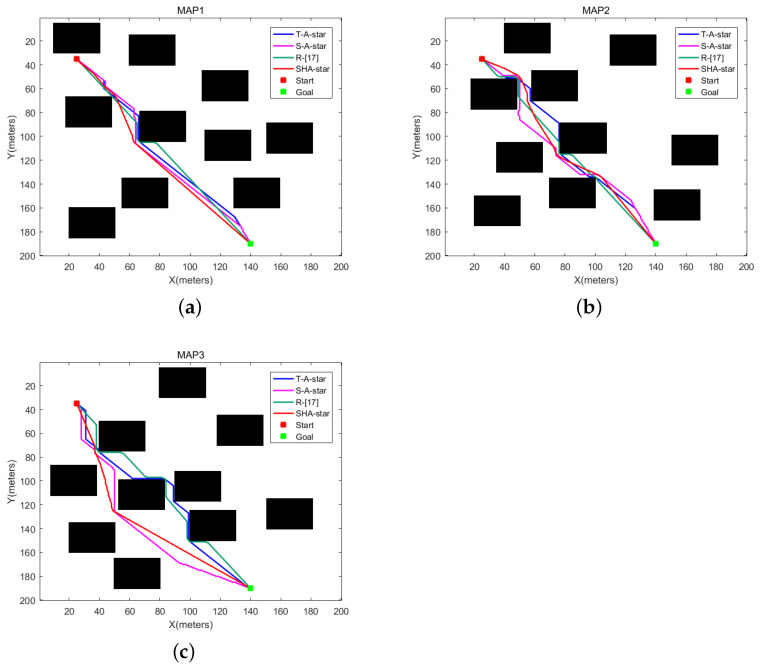
The second map-planning results. (**a**) Map 1, (**b**) Map 2, (**c**) Map 3.

The simulation results under the second category of environmental information are depicted in Figure 15. In this context, the red markers represent the starting points, the green markers signify the endpoints, and the different-colored lines within the graph represent the paths generated by T_A-star, S_A-star, R_[17], and SHA-star. All four algorithms successfully navigate from the starting point to the endpoint without encountering obstacles. Figure 15a–c reveal that within an environment characterized by randomly changing obstacles, SHA-star, by increasing the search freedom and integrating danger coefficients, effectively reduces the number of path inflection points. These paths maintain a certain safe distance from obstacles. In contrast, T_A-star, which does not account for path collision risk, leads to paths in close proximity to obstacles and features a higher number of inflection points due to its 8-directional search. Even with the incorporation of danger coefficients, S_A-star retains multiple inflection points. R_[17], due to its combination of linear and search paths, exhibits notably high path smoothness in Figure 15a–c. However, it too fails to consider collision risks during path planning, resulting in paths closely following obstacles.

Table 3 offers a comprehensive comparison of the four algorithms concerning path length, collision risk, search time, motion duration, path smoothness, and remaining power. The data clearly demonstrate that SHA-star excels by achieving the shortest paths, lowest collision risks, shorter runtime, smoother paths, and enhanced efficiency. While the search time increases slightly compared to T_A-star, SHA-star’s overall runtime is the shortest. When considering all aspects, SHA-star emerges as the superior choice in environments characterized by random obstacles.

### 4.3. Comparison of Irregular Obstacles

**Figure 16:**
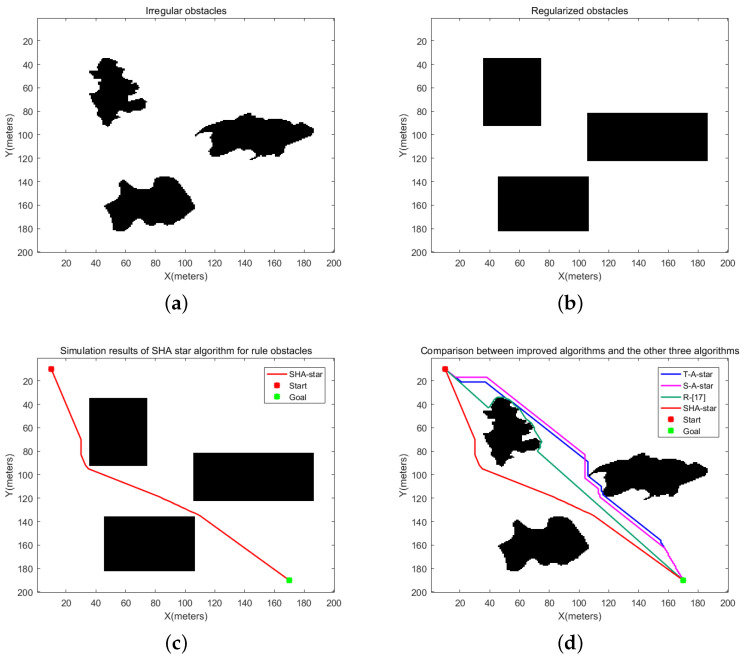
Simulation results of irregular obstacles. (**a**) Irregular obstacle environment, (**b**) Regularized obstacle environment, (**c**) SHA star algorithm simulation results, (**d**) simulation results of four algorithms.

To evaluate the performance of enhanced algorithms in handling environments with irregular obstacles, a comparative path-planning experiment was devised. In the experimental setup, the initial coordinates of the robot were set at (10,10), while the destination coordinates were set at (170,190). Figure 16a illustrates the layout of these irregular obstacles, while Figure 16b outlines the results after the regularization of the same obstacles. Figure 16c showcases the simulation outcomes of the improved algorithms within a context of regularized obstacles, whereas Figure 16d displays the paths generated by the other three algorithms within an environment featuring irregular obstacles, juxtaposed with the paths created by the SHA-star algorithm in the context of regularized obstacles. The relevant data are documented in Table 4.

Upon a close examination of the paths displayed in Figure 16d, it becomes evident that, post regularization of the obstacles, the paths obtained through the SHA-star algorithm maintain a considerable distance from the irregular obstacles. This characteristic significantly reduces collision risks. In contrast, the T_A-star algorithm and the R_[17] algorithm, which fail to account for the safety of robot navigation, produce paths that closely track the obstacles, resulting in a high susceptibility to collisions. The data in Table 5 further corroborate that SHA-star is capable of generating collision-free paths. In comparison to T_A-star, S_A-star, and R_[17], SHA-star’s optimal path length is reduced by 0.63%, 1.53%, and 3.16%, respectively. While regularizing the obstacles consumes some additional time, leading to a relatively longer path search time when compared to T_A-star and S_A-star, it still surpasses the search time required by R_[17]. Furthermore, SHA-star attains the shortest total runtime, even in this scenario. Additionally, SHA-star generates paths characterized by greater smoothness, decreasing path inflection points by 95.38%, 94.23%, and 84.62% in comparison to T_A-star, S_A-star, and R_[17]. The enhancement of path smoothness contributes to improved path continuity, eliminating the need for the robot to frequently change directions, thereby shortening the total runtime required for material-handling tasks. In conclusion, the paths created by the SHA-star algorithm retain the highest remaining energy.

**Figure 17:**
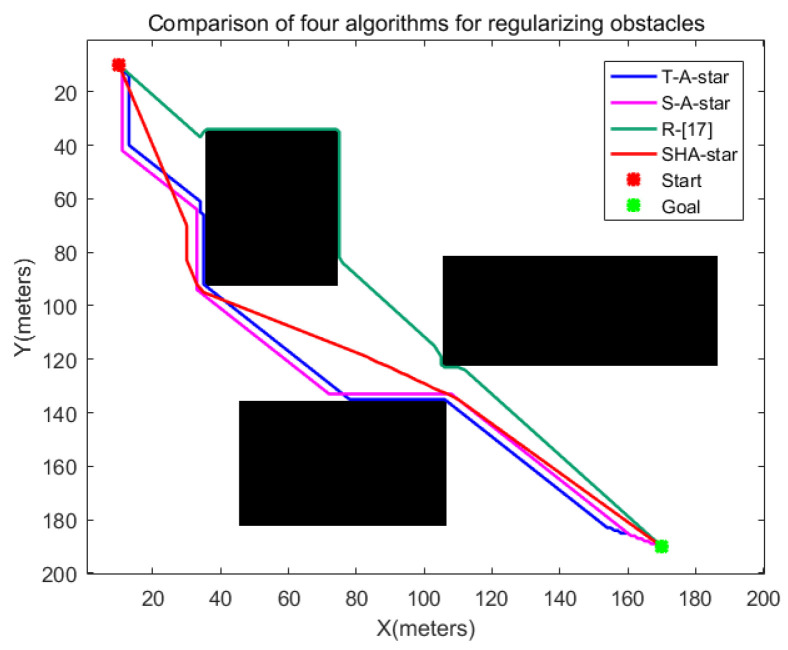
Simulation results of four algorithms in a regularized obstacle environment.

Figure 17 illustrates the simulation results of the four algorithms under regularized obstacles, with specific data comparisons presented in Table 5. From Figure 17, it is evident that without safety distance measures, the results obtained by T_A-star and R_[17] closely adhere to obstacles, while SHA-star and S_A-star algorithms yield safe paths. According to the data in Table 5, due to the increase in obstacle area after regularization, the path lengths obtained by T_A-star, R_[17], and S_A-star under regularized obstacles experience an increment. Moreover, since the regularization of obstacles requires a certain amount of time, the path search times for these three algorithms are greater than those for paths under irregular obstacles. In the remaining performance metric comparisons, the SHA-star algorithm outperforms T_A-star, R_[17], and S_A-star.

Considering these multifaceted factors, the SHA-star algorithm demonstrates outstanding path-planning performance in environments with irregular obstacles.

### 4.4. Simulation Comparison of Warehouse Storage Area

**Figure 18:**
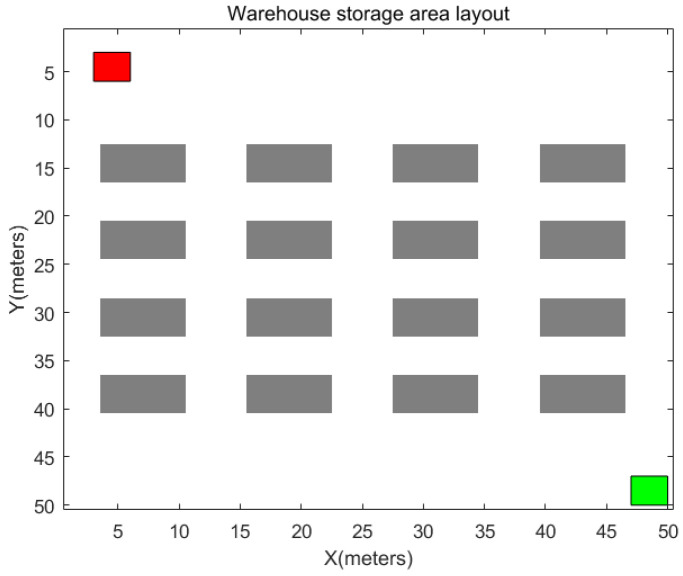
Map of cargo storage area.

**Figure 19:**
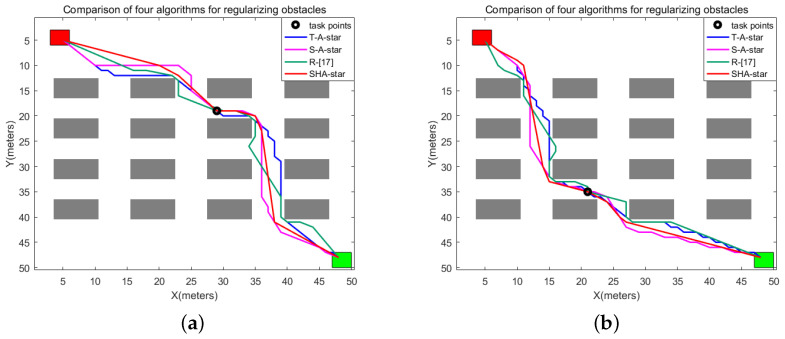
Comparison of simulation results. (**a**) Target Location 1. (**b**) Target Location 2.

Throughout the entire logistics process, spanning from the entry of goods into the warehouse to their final dispatch, a sequence of crucial steps encompasses key facets such as receiving, storage, picking, packing, and shipping. Typically, warehousing systems partition the facility into two main segments, one exclusively designated for the storage of goods and the other tailored for merchants, encompassing areas such as receiving and dispatch zones [26]. This paper centers its attention on the storage area, presuming its total area to be 50 × 50 m as illustrated in the designed model in Figure 18. The gray region in the figure represents shelves, the red area signifies the robot parking zone where the robot docks when not engaged in handling tasks, and the green area serves as the exit. Upon receiving instructions, the robot maneuvers from the parking zone to the specified shelf for cargo handling, subsequently transporting the goods to the exit. This study sets the target positions for cargo handling at (29, 19) and (21, 35). When computing time, the loading and unloading times are disregarded, with the focus solely being on the planning time and robot travel time. The simulation results, as depicted in Figure 19, encompass comparative data regarding path length, collision risk, path search time, operation time, smoothness, and remaining battery capacity in Table 6.

The simulation graphs allow us to observe that all four algorithms have successfully determined paths for the handling tasks. From the data presented in Table 6, it is evident that, across different task points, the SHA-star algorithm showcases a minimum reduction of 1.26% in path length, a significant decrease of 41.14% in collision risk, and a minimum improvement of 54.6% in path smoothness, concurrently minimizing energy consumption by 1.16%, when compared to the T_A-star, S_A-star, and R_[17] algorithms. Although the path search time is increased by 0.06 s compared to T_A-star, the overall path operation time remains the shortest.

The above simulation results and data analysis affirm that SHA-star can be effectively applied in warehousing scenarios. Furthermore, in the context of warehouse handling, the SHA-star algorithm consistently outperforms the other three algorithms as evidenced by the superior performance metrics.

### 4.5. Comparison with Multi-Objective Optimization Algorithms

**Figure 20:**
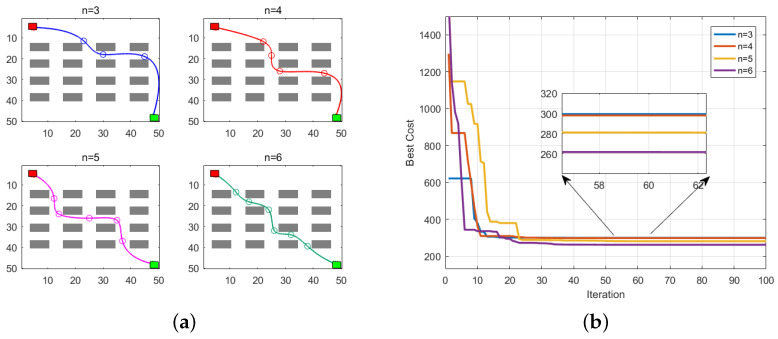
Simulation results under different control points of CAPSO. (**a**) Simulation result, (**b**) iterative curve.

In order to comprehensively assess the superiority of the improved A-star algorithm, this study elected to conduct a further comparative analysis involving particle swarm optimization (PSO) and genetic algorithm (GA). The algorithms from references [18] (CAPSO) and [19] (GA) were chosen as the benchmark algorithms for replication. Reference [18] underscored the impact of the number of control points on path planning. The experimental evidence suggests that a range of three to six control points produced favorable results. Consequently, in this investigation, a population size of 30 was chosen, and the number of control points was set between three and six. This experiment was repeated 50 times to establish an average outcome. The results are depicted in Figure 20, where Figure 20a showcases the paths planned by the CAPSO algorithm under various control point configurations, and Figure 20b illustrates the iteration curves for different control points. The outcomes indicate that the optimal results are achieved when six control points are employed.

Consequently, this study fixed the number of control points at 6, mimicking the parameters of literature [18], and used a population size of 30 and 100 iterations, with all other parameters consistent with literature [18]. For literature [19], the number of control points was also set at 6, with a population size of 30, and 100 iterations, with the remaining parameters consistent with literature [19].

Both of these algorithms were then applied to optimize multiple objectives, such as path length, collision risk, runtime, path smoothness, and energy consumption. However, in practical applications, certain performance metrics may lead to conflicts in robot path optimization. Additionally, the mentioned five indicators are on different orders of magnitude. To balance the impact of different objective functions, a single-objective standardization method [27] was adopted to select suitable weighting coefficients. The fitness function was defined as follows:

(27) $$\begin{array}{c} f=k_{1}L\left(s\right)+k_{2}R\left(S_{robot},S_{obs}\right)+k_{3}T+k_{4}A\left(S\right)-k_{5}E \end{array}$$

wherein one of the five weighting coefficients was set to 1, while the others were set to 0 for simulation experiments to obtain the minimum path length $L_{min}$, minimum collision risk $R_{min}$, minimum runtime $T_{min}$, minimum angle $A_{min}$, and maximum remaining energy $E_{max}$. Let $k_{1}=1$, then $k_{2}=L_{min}/R_{min}$, $k_{3}=L_{min}/T_{min}$, $k_{4}=L_{min}/A_{min}$, and $k_{5}=L_{min}/E_{max}$. The weighting coefficient selection for literature [18] and literature [19] is presented in Table 7. Due to the minimum collision risk $R_{min}=0$ obtained by the two algorithms, $k_{2}$ is set to 100.

**Figure 21:**
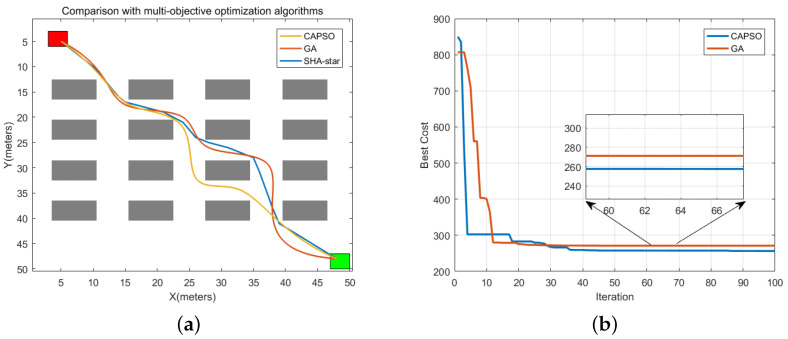
Comparison of simulation results between SHA-star, CAPSO, and GA. (**a**) Simulation result, (**b**) iterative curves of CAPSO and GA.

Figure 21a showcases the paths planned by CAPSO, GA, and the improved A-star algorithm, with the corresponding data recorded in Table 8. Moreover, Figure 21b visually illustrates the changes in fitness function values after 100 iterations for CAPSO and GA.

From Figure 21, it is evident that CAPSO, GA, and SHA-star algorithms can all identify optimal paths while maintaining a certain safe distance from obstacles. Based on the data analysis in Table 8, it can be concluded that the CAPSO algorithm outperforms SHA-star in terms of path length, collision risk, and operation time. In terms of smoothness, SHA-star exhibits lower smoothness due to the straight-line connections in the latter part of the planned path, resulting in fewer corners. Consequently, CAPSO surpasses SHA-star in terms of smoothness. Additionally, SHA-star excels over CAPSO in remaining battery capacity.

The GA algorithm demonstrates lower collision risk than SHA-star, but when compared to SHA-star, it lags behind in path length, operation time, path smoothness, and remaining energy. Notably, after 50 simulation experiments with averaged results, SHA-star’s path search time is merely 0.054 s, whereas the CAPSO and GA algorithms require a substantial amount of time for path searching. This extended search time significantly diminishes path-planning efficiency.

Based on the data analysis above, it can be concluded that CAPSO and GA are more suitable for scenarios with strict collision risk requirements and lenient planning time requirements. In a warehousing environment, considerations extend beyond collision risk to encompass planning time, path smoothness, and more. Therefore, taking a holistic perspective, the SHA-star algorithm proves to be more suitable for path planning in warehousing environments.

## 5. Conclusions

This study introduces a search strategy-based safety heuristic approach to address challenges in traditional robot path planning. Leveraging risk coefficients to select safe paths and employing an environmental partitioning strategy, this method aims to overcome limitations inherent in conventional algorithms. Extensive simulation experiments consistently validate its capability to generate safe and smooth paths across various map environments, effectively enhancing both the operational efficiency and safety of the robot.

The core algorithm in this research is the A-star algorithm, and while it has undergone significant enhancements, we acknowledge that there is room for further improvement. Firstly, a major drawback of the A-star algorithm lies in its exponential space growth. Recognizing substantial room for improvement in this regard, we commit to ongoing research to overcome this issue. Secondly, this study employs a relatively simplified robot motion model, not fully considering the robot’s dynamic model. Such simplification may lead to decreased path-planning accuracy in certain complex scenarios. Future research directions will involve introducing more precise robot dynamic models to enhance path accuracy and feasibility. Additionally, in dealing with environments filled with irregular obstacles, the obstacle normalization process consumes a considerable amount of time. In future research, we will strive to identify more efficient methods to handle such irregular obstacles, further improving the efficiency of path planning.

## Figures and Tables

**Table 1:** Comparison of the experimental data for the three distance formulas.

Method	Manhattan	Euclidean	Chebyshev
Length/m	70	61.799	61.799
Turning point	6	5	11

**Table 2:** The first kind of map-planning data.

Map	Path Parameters	T_A-Star	S_A-Star	R_[17]	SHA-Star
Convex obstacle	Path length/m	380.5584	384.0731	446.3637	369.5521
	$P_{length}$	2.89%	3.78%	17.21%	-
	Collision risk value	309.0022	18.1278	674.88	10.6796
	$P_{collision}$	96.54%	41.09%	98.42%	-
	Searching time/s	0.821	1.725	1.638	1.084
	$P_{S\_time}$	−12.03%	37.16%	33.82%	-
	Run time/s	408.99	412.79	479.74	397.18
	$P_{R\_time}$	2.89%	3.78%	17.21%	-
	Smoothness/radian	89.5353	89.5353	15.3482	6.1191
	$P_{smoothness}$	93.17%	93.17%	60.13%	-
	Residual power	47.69%	47.34%	52.92%	62.07%
	$P_{power}$	27.49%	27.97%	19.44%	-
Narrow direction of leg gap	Path length/m	421.7056	426.8772	557.1755	414.3527
	$P_{length}$	1.74%	2.93%	25.63%	-
	Collision risk value	1032.9641	121.5572	1052.6305	12.5214
	$P_{collision}$	98.79%	89.70%	98.81%	-
	Searching time/s	0.746	1.988	1.758	1.322
	$P_{S\_time}$	−17.21%	33.50%	24.80%	-
	Run time/s	453.26	458.82	598.87	445.3636
	$P_{R\_time}$	1.74%	2.93%	25.63%	-
	Smoothness/radian	67.5442	64.4026	19.1333	8.2937
	$P_{smoothness}$	87.72%	87.12%	56.65%	-
	Residual power	47.07%	47.06%	41.23%	57.24%
	$P_{power}$	19.21%	19.23%	27.24%	-

**Table 3:** The second kind of map-planning data.

Map	Path Parameters	T_A-Star	S_A-Star	R_[17]	SHA-Star
Map 1	Path length/m	202.6345	202.6345	200.2812	196.5703
	$P_{length}$	2.99%	2.99%	1.85%	-
	Collision risk value	144.3658	0	113.4513	0
	$P_{collision}$	100.00%	-	100.00%	-
	Searching time/s	0.444	1.921	1.758	1.662
	$P_{S\_time}$	−174.32%	13.48%	5.46%	-
	Run time/s	217.80	217.63	215.27	211.27
	$P_{R\_time}$	3.00%	2.92%	1.86%	-
	Smoothness/radian	18.8495	14.1371	3.1433	2.1136
	$P_{smoothness}$	88.79%	85.05%	32.76%	-
	Residual power	76.73%	77.48%	79.47%	80.00%
	$P_{power}$	14.05%	11.19%	2.58%	-
Map 2	Path length/m	209.0782	215.1787	205.7177	202.2907
	$P_{length}$	3.25%	5.99%	1.67%	-
	Collision risk value	319.2708	74.6585	241.0164	0.493
	$P_{collision}$	99.85%	99.34%	99.80%	-
	Searching time/s	0.432	1.571	1.467	1.441
	$P_{S\_time}$	−133.56%	8.27%	1.77%	-
	Run time/s	224.73	231.10	221.12	217.42
	$P_{R\_time}$	3.25%	5.92%	1.67%	-
	Smoothness/radian	26.7035	34.5575	8.4618	3.9394
	$P_{smoothness}$	85.25%	88.60%	53.44%	-
	Residual power	74.84%	72.98%	78.08%	79.14%
	$P_{power}$	17.09%	22.80%	4.84%	-
Map 3	Path length/m	215.5218	217.865	217.815	206.8147
	$P_{length}$	4.04%	5.07%	5.05%	-
	Collision risk value	402.6708	0	383.6158	0
	$P_{collision}$	100.00%	-	100.00%	-
	Searching time/s	0.569	1.998	2.047	1.669
	$P_{S\_time}$	−193.32%	16.47%	18.47%	-
	Run time/s	231.65	233.99	234.12	222.28
	$P_{R\_time}$	4.04%	5.00%	5.05%	-
	Smoothness/radian	9.4247	40.8407	9.3452	6.4203
	$P_{smoothness}$	31.88%	84.28%	31.30%	-
	Residual power	76.94%	71.71%	76.73%	78.29%
	$P_{power}$	5.85%	23.26%	6.70%	-

**Table 4:** Comparison data of irregular obstacles.

Map	Path Parameters	T_A-Star	S_A-Star	R_[17]	SHA-Star
Irregular obstacles	Path length/m	256.2325	258.5756	262.9066	254.6071
	$P_{length}$	0.63%	1.53%	3.16%	-
	Collision risk value	93.3621	0	141.0502	0
	$P_{collision}$	100.00%	-	100.00%	-
	Searching time/s	0.622	2.181	4.367	2.639
	$P_{S\_time}$	−124.28%	−11.00%	39.57%	-
	Run time/s	275.41	277.71	282.58	273.65
	$P_{R\_time}$	0.64%	1.46%	3.16%	-
	Smoothness/radian	31.4159	25.1327	9.4315	1.4507
	$P_{smoothness}$	95.38%	94.23%	84.62%	-
	Residual power	69.37%	70.14%	72.20%	73.90%
	$P_{power}$	14.79%	12.59%	6.12%	-

**Table 5:** The simulation data of the four algorithms under the regular obstacles.

Map	Path Parameters	T_A-Star	S_A-Star	R_[17]	SHA-Star
Four algorithms for data under regularized obstacles	Path length/m	267.9482	270.2914	272.8028	254.6071
	$P_{length}$	4.98%	5.80%	6.67%	-
	Collision risk value	361.0685	0	579.6919	0
	$P_{collision}$	100.00%	-	100.00%	-
	Searching time/s	1.482	2.952	5.291	2.639
	$P_{S\_time}$	−18.07%	10.60%	50.12%	-
	Run time/s	288.00	290.29	293.22	273.65
	$P_{R\_time}$	4.98%	5.73%	6.67%	-
	Smoothness/radian	18.8495	10.9955	8.4829	1.4507
	$P_{smoothness}$	92.30%	86.81%	82.90%	-
	Residual power	70.20%	71.22%	71.36%	73.90%
	$P_{power}$	12.42%	9.31%	8.87%	-

**Table 6:** Planning data in the warehouse environment.

Map	Path Parameters	T_A-Star	S_A-Star	R_[17]	SHA-Star
Location of goods: (29, 19)	Path length/m	69.5979	72.0951	72.8876	68.7244
	$P_{length}$	1.26%	4.68%	5.71%	-
	Collision risk value	138.1442	42.4914	113.3893	25.0093
	$P_{collision}$	81.90%	41.14%	77.94%	-
	Searching time/s	0.005	0.068	0.308	0.059
	$P_{S\_time}$	−1080.0%	11.76%	80.52%	-
	Run time/s	74.81	77.43	78.34	73.86
	$P_{R\_time}$	1.26%	4.60%	5.72%	-
	Smoothness/radian	10.9955	8.1757	15.2596	3.7121
	$P_{smoothness}$	66.24%	54.60%	75.67%	-
	Residual power	91.29%	91.48%	90.28%	92.53%
	$P_{power}$	14.24%	12.32%	23.15%	-
Location of goods: (21, 35)	Path length/m	70.7695	69.4984	71.3202	68.7627
	$P_{length}$	2.84%	1.06%	3.59%	-
	Collision risk value	127.4218	43.8785	139.0656	15.2428
	$P_{collision}$	88.04%	65.26%	89.04%	-
	Searching time/s	0.006	0.076	0.359	0.066
	$P_{S\_time}$	−1000.0%	13.16%	81.62%	-
	Run time/s	76.07	74.64	76.66	73.91
	$P_{R\_time}$	2.84%	0.98%	3.59%	-
	Smoothness/radian	25.1327	9.6706	11.3625	3.4535
	$P_{smoothness}$	86.26%	64.29%	69.61%	-
	Residual power	88.92%	91.51%	91.05%	92.57%
	$P_{power}$	32.94%	12.49%	16.98%	-

**Table 7:** Weight coefficient value.

	$k_{1}$	$k_{2}$	$k_{3}$	$k_{4}$	$k_{5}$
CAPSO	1	100	0.94	16.76	0.69
GA	1	100	0.97	10.36	0.75

**Table 8:** Comparative data between SHA-star and PSO algorithms.

Path Parameters	CAPSO	GA	SHA-Star
Path length/m	64.53	67.98	64.93
Collision risk value	0	0	19.87
Path-searching time/s	19.45	20.82	0.054
Run time/s	68.25	69.95	69.79
Smoothness/radian	4.745	6.562	3.853
Residual power	92.37%	91.16%	92.53%

## Data Availability

Data are contained within the article.
